# Intrauterine Shaping of Fetal Microbiota

**DOI:** 10.3390/jcm13175331

**Published:** 2024-09-09

**Authors:** Norbert Dera, Natalia Żeber-Lubecka, Michał Ciebiera, Katarzyna Kosińska-Kaczyńska, Iwona Szymusik, Diana Massalska, Kacper Dera, Katarzyna Bubień

**Affiliations:** 1 Department of Obstetrics, Perinatology and Neonatology, Center of Postgraduate Medical Education, 01-809 Warsaw, Poland; katarzyna.kosinska-kaczynska@cmkp.edu.pl (K.K.-K.); iwona.szymusik@gmail.com (I.S.); k.bubien@gmail.com (K.B.); 2 Warsaw Institute of Women’s Health, 00-189 Warsaw, Poland; michal.ciebiera@gmail.com (M.C.); diana_massalska@wp.pl (D.M.); 3 Department of Gastroenterology, Hepatology and Clinical Oncology, Centre of Postgraduate Medical Education, 02-781 Warsaw, Poland; natalia.zeber-lubecka@cmkp.edu.pl; 4 Department of Genetics, Maria Sklodowska-Curie National Research Institute of Oncology, 02-781 Warsaw, Poland; 5 Second Department of Obstetrics and Gynecology, Centre of Postgraduate Medical Education, 00-189 Warsaw, Poland; 6 Provincial Specialist Hospital in Olsztyn, 10-561 Olsztyn, Poland; kacper.dera@interia.pl

**Keywords:** colonization, microbiome, microbiota, dysbiosis, transmission, fetal microbiota, newborn, premature infant, vagina, uterus

## Abstract

Mechanisms resulting from the physiological immaturity of the digestive system in children delivered before 32 weeks of gestation and, in particular, different interactions between the microbiome and the body have not been fully elucidated yet. Next-generation sequencing methods demonstrated the presence of bacterial DNA in the placenta and amniotic fluid, which may reflect bacterial populations that initiate intestinal colonization in utero. Numerous studies confirmed the hypothesis stating that intestinal bacteria played an important role in the pathogenesis of necrotizing enterocolitis (NEC) early- and late-onset neonatal sepsis (EONS and LONS). The model and scale of disorders within the intestinal microbiome are the subject of active research in premature infants. Neonatal meconium was primarily used as an indicator defining the environment in utero, as it is formed before birth. Metagenomic results and previous data from microbiological bacterial cultures showed a correlation between the time from birth to sample collection and the detection of bacteria in the neonatal meconium. Therefore, it may be determined that the colonization of the newborn’s intestines is influenced by numerous factors, which may be divided into prenatal, perinatal, and postnatal, with particular emphasis put on the mode of delivery and contact with the parent immediately after birth. **Background**: The aim of this review was to collect available data on the intrauterine shaping of the fetal microbiota. **Methods**: On 13 March 2024, the available literature in the PubMed National Library of Medicine search engine was reviewed using the following selected keywords: “placental microbiome”, “intestinal bacteria in newborns and premature infants”, and “intrauterine microbiota”. **Results**: After reviewing the available articles and abstracts and an in-depth analysis of their content, over 100 articles were selected for detailed elaboration. We focused on the origin of microorganisms shaping the microbiota of newborns. We also described the types of bacteria that made up the intrauterine microbiota and the intestinal microbiota of newborns. **Conclusions**: The data presented in the review on the microbiome of both term newborns and those with a body weight below 1200 g indicate a possible intrauterine colonization of the fetus depending on the duration of pregnancy. The colonization occurs both via the vaginal and intestinal route (hematogenous route). However, there are differences in the demonstrated representatives of various types of bacteria, phyla *Firmicutes* and *Actinobacteria* in particular, taking account of the distribution in their abundance in the individual groups of pregnancy duration. Simultaneously, the distribution of the phyla *Actinobacteria* and *Proteobacteria* is consistent. Considering the duration of pregnancy, it may also be concluded that the bacterial flora of vaginal origin dominates in preterm newborns, while the flora of intestinal origin dominates in term newborns. This might explain the role of bacterial and infectious factors in inducing premature birth with the rupture of fetal membranes.

## 1. Introduction

The aim of this review was to collect available data on the intrauterine shaping of the fetal microbiota. In the first weeks and months of life, gut microbiome disorders affect the infant’s growth, development, and health status. Impaired intestinal microbiota formation during infant development results in an increased risk of immune and metabolic diseases that may persist in childhood and, potentially, continue into adulthood [1]. Most research on the development of the intestinal microbiome focused on term infants [2]. The physiological immaturity of the digestive system in infants delivered before 32 weeks of gestation was found to lead to several interactions between the microbiome and the body. The underlying mechanisms have not been fully elucidated yet [3].

The maturation of both intestinal barrier function and immunity occurs in the prenatal period. The fetal intestine is more permeable to macromolecules and less tolerant of antigens compared to the infant intestine after birth. The transfer of maternal immunoglobulin G (IgG) through the placenta and the absorption in the fetal intestine increase in the period close to the term of pregnancy, shaping the immune response of the newborn after birth [4]. Therefore, the intrauterine environment is able to shape health far beyond the fetal life and may influence long-term changes in health parameters. According to recent research, colonization with specific microorganisms in early life may predispose to the development of childhood diseases, including asthma and obesity [5,6]. Several teams of researchers, using methods based on next-generation sequencing, showed the presence of bacterial DNA in the placenta and amniotic fluid [7,8]. It was also suggested that it reflected bacterial populations that initiated intestinal colonization in utero. According to Bartnicka et al., the use of the latest molecular techniques allowed the confirmation of the presence of bacteria in the amniotic fluid, umbilical cord blood, placenta, and fetal membranes. In addition, the detection of individual bacterial species in the meconium (such as *Escherichia coli*, *Enterococcus faecium*, and *Staphylococcus epidermidis*) might result from their translocation via maternal bloodstream. The translocation hypothesis was confirmed by the fact that bacteria of the genera *Enterococcus*, *Streptococcus*, *Staphylococcus*, and the *Propionibacterium* species were isolated from the umbilical cord blood [9].

Although the presence and function of the placental microbiota have not been fully elucidated, it is known that the intestinal microflora during pregnancy is a key determinant of the health of the offspring. According to abundant evidence, the maternal intestinal microbiota during pregnancy might determine the development of atopy and autoimmune phenotypes in the offspring and affect the child’s immunity and predisposition to an occurrence of disease [10].

Two conceptual models were used to describe the development of the intestinal microbiome of premature infants. According to the first concept, the intestinal microbiome developed in an orderly manner, depending on the maturation of the child’s body, with the minor impact of environmental factors. The second concept assumed that external factors such as the diet, exposure to antibiotics, and the hospital environment played a major role in the development of the microbiome [11].

Postnatally, in early life, a balanced gut microbiome is crucial for the functioning of the body and the maturation of the immune system [12]. In recent years, a growing body of evidence has pointed to the importance of the gut microbiome in providing resistance to colonization by pathogens or opportunistic intestinal pathobionts. Disorders of intestinal microbiota maturation, i.e., intestinal dysbiosis, predispose newborns to the formation of NEC [13], nosocomial infections, or EONS and LONS [14,15,16,17]. 

Low weight gain in premature infants was found to be associated with disorders in the gut–brain axis [18], the composition of the intestinal microbiota [19,20], insufficient absorption of nutrients [21], and the existing diseases of neonatal age. Numerous studies confirmed the hypothesis that intestinal bacteria played an important role in the pathogenesis of NEC, EONS, and LONS. However, it was impossible to clearly identify one bacterial species as the causative agent. Therefore, in the case of NEC, an increase in the abundance of several microorganisms was observed, mainly from the phyla *Firmicutes* and *Proteobacteria* (*Cronobacter sakazakii*, *Klebsiella* sp., and *E. coli*) [22,23]. An increased abundance of *Clostridium* spp. and their toxins was detected in the stool of neonates with NEC [24,25,26].

**Figure 1:**
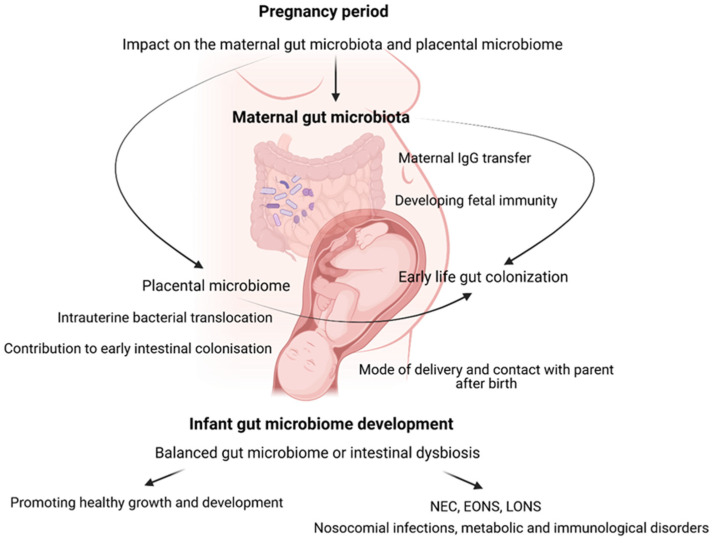
Complex interactions and outcomes related to the infant gut microbiome. During pregnancy, maternal gut microbiota and the placental microbiome influence fetal immunity through maternal IgG transfer [4]. Intrauterine bacterial translocation contributes to early-life gut colonization, shaping the infant gut microbiome. This microbiome development may lead to either a balanced gut microbiome, promoting proper functioning, immunity, and healthy growth, or intestinal dysbiosis, which is associated with conditions like NEC, EONS, LONS, nosocomial infections, and metabolic and immune disorders. Factors such as prenatal conditions, mode of delivery, and immediate postnatal contact with parents significantly affect microbiome development and subsequent infant health outcomes.

Importantly, the colonization of the newborn’s intestine was found to be influenced by numerous factors, which might be divided into prenatal, perinatal, and postnatal, with particular emphasis put on the mode of delivery and contact with the parent immediately after birth [27,28] (Figure 1).

This review focuses on prenatal factors affecting the course of pregnancy and the condition of the fetus. They underlie the proper functioning in later life. Over ten times more bacteria than cells are found in the human body [29,30]. Sequencing the hypervariable regions of the bacterial 16S RNA gene enabled the taxonomic identification of bacteria at the level of the genus and species, but not at the subspecies level [31]. Shotgun metagenomics could identify bacteria at a lower level of taxonomic resolution, which means the detection of low- or very low-abundance microbial communities and could also be more effective in differentiating closely related species [32]. The development of next-generation sequencing made it possible to obtain an image of the reproductive microbiome. In 2015, Franasiak et al. were the first to determine the microbiota of the uterine cavity based on material collected from the end of the catheter during embryo transfer [31]. 

Various hypotheses regarding the origin of the microbiota of the uterus referred both to the pathway ascending from the vagina and the intestinal tract (through the filtration of the intestinal vessels into the peritoneal cavity with reabsorption through the fallopian tubes, and with the help of dendritic cells and leukocytes transporting hematogenous material to the uterus) [33]. DiGiulio et al. determined that the amniotic fluid from premature births could be colonized by the placental microbiome and by microorganisms ascending from the vagina, while maintaining the continuity of the amniotic membrane [34]. Some authors suggested that the vagina might be the source of microbes that reached the placenta, amniotic fluid, and fetus via translocation through the chorion [35,36]. It was also confirmed that, during pregnancy, the microbiota of the mother’s vagina affected the immunity of the fetus in the uterus, even before passing through the vaginal canal during childbirth.

Until 2014, it had been believed that the placenta had not contained its own microbiome. This was related to the research techniques used, which had not taken account of differences between live and dead bacteria and had not allowed for obtaining a maternal blood sample facilitating the determination whether the microbiome came from the maternal chorion or the fetus. Therefore, the results were questionable [37]. However, according to current research, infections are suggested to underlie PPROM and preterm birth [38,39,40]. The placenta was demonstrated to have its own healthy microbiome, mainly containing *Firmicutes*, *Tenericutes*, *Proteobacteria*, *Bacteroidetes*, and *Fusobacteria*. It was also found that the placental microbiome was the most similar to the human oral microbiome [41].

Establishing and maintaining the integrity and function of the placenta is critical to fetal growth, development, and survival [42]. In a cross-sectional study including 195 patients, Stout et al. demonstrated that intracellular Gram-positive and Gram-negative bacteria were present in the basal plate (a structure including a layer of tissue directly at the maternal-fetal interface and below).

## 2. Materials and Methods

On 13 March 2024, the available literature in the PubMed National Library of Medicine search engine was reviewed, using the following selected keywords: “placental microbiome”, “intestinal bacteria in newborns and premature infants”, and “intrauterine microbiota”. After reviewing the available articles and abstracts and an in-depth analysis of their content, over 100 articles were selected for detailed elaboration. We focused on the origin of microorganisms shaping the microbiota of newborns. We also described the types of bacteria that made up the intrauterine microbiota and the intestinal microbiota of newborns.

## 3. Discussion

### 3.1. Differentiation of Views—Sterile (Acquired at Birth)/Non-Sterile Environment

The present literature review draws attention to the evolution of views regarding the uterine environment. The original views assumed that the cervical mucus, which adhered to the vagina colonized by bacteria, maintained uterine sterility. At the same time, in 1996, Egbase et al. claimed that the microflora of the reproductive system might affect the results of in vitro fertilization [43]. In subsequent years and in the latest research, the above hypotheses regarding uterine sterility were repeatedly undermined [44,45,46,47]. The only differences consisted in the way and method of the colonization of the uterus and the most identified bacterial flora. The examination of the neonatal meconium constitutes an indirect confirmation of the lack of sterility of the uterine cavity. According to Jimmy Kok-Foo Lee et al., over 50% of the meconium in the population of preterm newborns contained bacteria, and the percentage increased with gestational age, which indicated the intrauterine acquisition of the bacterial flora [11]. This is an extremely important aspect in relation to the functioning of the extrauterine human body. It constitutes the basis for the analysis and identification of the adequate bacterial flora of the gastrointestinal tract as a protective factor that influences the proper development and functioning from the neonatal period to adulthood.

### 3.2. The Way of the Colonization of the Uterus: By Continuity, by Blood

According to the latest hypothesis, the uterine microbiota may ascend from the vagina and the intestinal tract. It occurs through the filtration of the intestinal vessels into the peritoneal cavity with reabsorption through the fallopian tubes and with the help of dendritic cells and leukocytes transporting hematogenous material to the uterus [33]. Jimenez et al. conducted animal studies and reported that after placing the genetically labeled *Enterococcus faecium* in the oral cavity of mice, it could be detected in the placenta. Similarly, they isolated the labeled *Enterococcus faecium* from the meconium of offspring after the oral inoculation of the strain to their pregnant mothers. It confirmed the above assumption that the intestinal microorganisms of mothers potentially penetrated the placenta into the intestines of their offspring [48].

Conversely, Goldenberg and Hanley suggested that the vagina might be the source of microbes that reached the placenta, amniotic fluid, and fetus via translocation through the chorion and amnion [35,36].

DiGiulio et al. determined that the amniotic fluid from premature births could be colonized by microorganisms ascending from the vagina while maintaining the continuity of the amniotic membrane [34]. The placental microbiome could also contribute to the colonization, which might be associated with the possible hematogenous translocation of the microbiome originating from the maternal oral cavity [41,49]. According to DiGiulio, species that had been long implicated in causing microbial invasion of the amniotic cavity (MIAC) remained among the common invaders (e.g., *Ureaplasma* spp., *Mycoplasma* spp., *Fusobacterium* spp., *Streptococcus* spp., *Bacteroides* spp., and *Prevotella* spp.). Similarly, cultivation-resistant anaerobes belonging to the family *Fusobacteriaceae* (particularly *Sneathia sanguinegens* and *Leptotrichia* spp.) and *Candida* spp. were commonly found in amniotic fluid [34]. Microbial invasion into the uterus was confirmed in 25–40% of preterm births [50,51], and in 7–12% of preterm births with intact membranes [52]. In the case of fetal membrane inflammation, the inflammatory process of the fetal surface of the placenta, the most frequently isolated pathogens included the following bacteria: *Bacteroides*, *E. coli*, *Gardnerella vaginalis*, *Mycoplasma hominis*, *Peptostreptococci*, *Streptococci*, and *Ureaplasma urealyticum* [53]. Therefore, it may be assumed that pathological bacteria might enter the amnion and chorion from the vagina. Some authors also hypothesized the transmission of an infection through the placenta from the oral cavity of patients with periodontal diseases [54]. Specific pathogenic bacteria of the oral cavity, including *Fusobacterium nucleatum*, *Porphyromonas gingivalis*, *Filifactor alocis*, and *Campylobacter rectus*, were associated with both periodontitis and the development of pregnancy diseases [54].

### 3.3. The Microbiota of the Oral Cavity and the Placenta of a Pregnant Woman

Research comparing the oral and placental microbiota performed on murine models also confirmed the similarity of the above locations. This may indicate the hematogenous origin of the placental microbiome [55,56,57,58]. The above was confirmed by the results of studies by Han et al., who found that a single *Bergeyella* strain was 100% identical at the 16S–23S rRNA sequence level between the patient’s periodontal subgingival plaque and the amniotic fluid. Both samples were 99.7% identical to the previously deposited oral *Bergeyella* sequence. None of the numerous vaginal samples collected from the patient revealed any *Bergeyella* species, and it was not known whether *Bergeyella* was commensal to any part of the body except the oral cavity [56]. Jimenez et al. demonstrated that the genetically labeled *Enterococcus faecium* placed in the oral cavity of mice could be later detected in the placenta. Importantly, it may provide numerous possibilities in the evaluation and modification of the bacterial flora of the maternal gastrointestinal tract. Considering the hematogenous origin of the placental microbiome, the proper supplementation of specific bacterial cultures in pregnant women might modulate the bacterial flora of the fetus and newborn with beneficial microbiome that might protect against colonization with harmful microorganisms.

### 3.4. Maternal Placental and Uterine Microbiome

Studies using the high-throughput sequencing technology confirmed the presence of the placental microbiome [59,60]. As regards the types of bacteria occurring in the placenta, Stout et al. demonstrated that both intracellular Gram-positive and Gram-negative bacteria were present in the basal plate (a structure including a layer of tissue directly at the maternal-fetal interface and below). They were observed in almost one-third of placental samples, with a high incidence in preterm births <28 weeks of gestation, but independently of the clinical or pathological features of fetal membrane inflammation [61]. Suggestions regarding the origin of intrauterine infections associated with premature birth, referring to the onset in the lower genital tract and penetration into the “sterile” intrauterine environment [50,62] contradicted the results of research using DNA-based technology, indicating commensal species commonly found in the oral cavity as the main causative factor of colonization [55,56,57,58]. The above was confirmed by the finding that oral microorganisms such as *Fusobacterium nucleatum* (Gram-negative oral anaerobes) might facilitate the hematogenous transmission of other commensal bacteria, i.e., *Escherichia coli*, during placental formation. It is related to their capability of binding to the vascular endothelium and changing the permeability [63].

Studies assessing the predominance of some species conducted by Moore et al. and Aagaard et al. revealed that *E. coli* was the most common microorganism in the placenta [41,64]. Simultaneously, they identified additional species of the oral microbiome, including *Prevotella tannerae* (gingival fissures) and non-pathogenic *Neisseria* species (mucosal surfaces). The above might be confirmed by research by Gosalbes et al., who revealed the abundance of *E. coli* in the meconium [65]. Conversely, Aagaard et al. demonstrated that the placenta had its own healthy microbiome, which mainly contained *Firmicutes*, *Tenericutes*, *Proteobacteria*, *Bacteroidetes*, and *Fusobacteria*. Overall, the profiles of the placental microbiome were the most similar (the Bray–Curtis dissimilarity < 0.3) to the one of the human oral microbiome [41]. At the same time, the intestinal bacterial flora was discussed. It included both *Bacteroidetes* and *Firmicutes*, which constituted 90% of the microflora, but also less abundant types, i.e., *Proteobacteria*, *Actinobacteria*, *Fusobacteria*, and *Verrucomicrobia*. Anaerobes constitute over 99% of normal intestinal microorganisms inhabiting mainly the distal part of the ileum and the colon [66,67]. To compare, four main types of bacteria, i.e., *Firmicutes*, *Proteobacteria*, *Actinobacteria*, and *Bacteroidetes* were present in the intestines of healthy newborns and premature infants with very low birth weight (VLBW) [68,69,70,71,72]. Nevertheless, premature infants and VLBW infants were characterized by lower microbial diversity, disturbed intestinal microbiome, and an increased colonization of potentially pathogenic nosocomial microorganisms compared to age-matched term infants [73]. 

Research on the uterine microbiome of healthy women also confirmed that the most consistent types included *Firmicutes*, *Bacteroidetes*, *Proteobacteria*, and *Actinobacteria* [31,74,75,76,77,78,79,80,81,82]. Conversely, *Lactobacillus* and *Streptococcus*, which may be found in the vagina and cervix, were the most commonly reported [83]. 

Regarding the dependence of individual bacterial species on the location, Brotman et al. found that each location in the body was characterized by one or several characteristic types, i.e., *Firmicutes* in the vagina, *Actinobacteria* in the skin of the retroauricular fold and the anterior nostrils, *Proteobacteria* and *Firmicutes* at all sites in the oral cavity, and *Bacteroidetes* in the feces. The placental microbiome was characterized by a greater abundance of *Proteobacteria* and the unique presence of *Tenericutes*, including the known intrauterine types of *Mycoplasma* and *Ureaplasma* [84].

**Figure 2:**
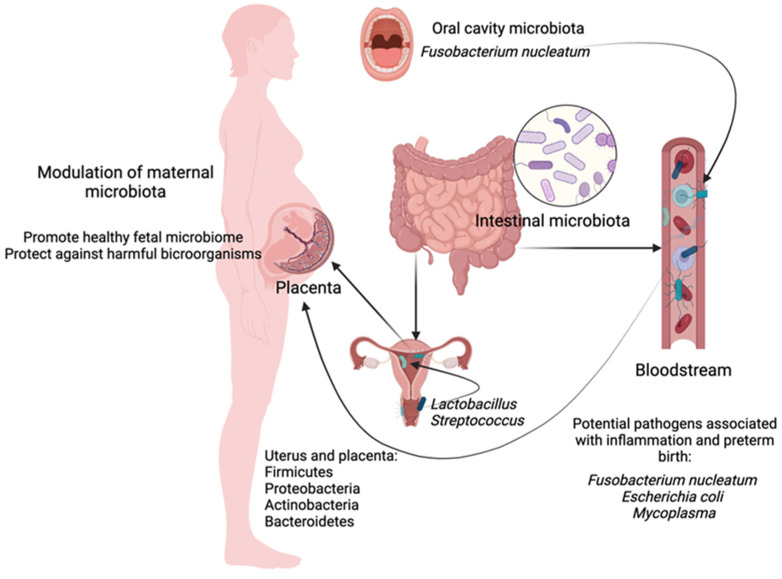
Colonization of the uterus and the relationship between the maternal microbiome and fetal development. Microbiota can ascend from the vagina and the intestinal tract through pathways including the peritoneal cavity and fallopian tubes, as well as through hematogenous routes. Microbes from the oral cavity and intestines can enter the bloodstream and reach the placenta, suggesting a hematogenous origin for the placental microbiome. The uterus is shown as a non-sterile environment, harboring common bacteria such as *Firmicutes*, *Proteobacteria*, *Actinobacteria*, and *Bacteroidetes*. Specific pathogens like *Fusobacterium nucleatum*, *Escherichia coli*, and *Mycoplasma* are linked to inflammation and preterm birth. Modulating the maternal microbiome through probiotics or dietary changes can influence the fetal microbiome positively, potentially protecting against harmful microorganisms. This highlights the importance of maternal microbiota in fetal development and the potential for interventions to promote a healthy pregnancy.

The above studies showed that the intrauterine environment was not “sterile”. The origin of the microbiota may largely be associated with hematogenous transmission rather than the assumed ascending transmission from the vagina. At the same time, there are four main types of bacteria in the intestines of a healthy newborn that are characteristic and present in the normal microbiome of the placenta and uterus of healthy women. Conversely, based on the analysis of the bacterial flora of preterm infant intestines, which are characterized by lower diversity, with an increased colonization of potentially pathogenic microorganisms, it may be concluded that the dysbiosis of maternal body, gastrointestinal tract and, consequently, the uterus and placenta, constitutes the basis of the inflammatory process being the main causative factor for premature birth. Therefore, such a relationship, i.e., the assessment of the maternal microbiota and its modulation, may provide a powerful protective tool for the development of the embryo and fetus (Figure 2).

### 3.5. Meconium Microbiota Depending on Gestational Age and Specific Diseases

The neonatal meconium is formed before birth. Therefore, it was primarily used as an indicator defining the in utero environment [85]. However, the analyses did not comprise acquired microorganisms that appear during and/or immediately after birth. Metagenomic results and previous data from bacterial cultures showed a correlation between the time from birth to sample collection and the detection of bacteria in the neonatal meconium.

Studies in mice and humans revealed that the meconium was colonized by bacteria during pregnancy [14,86,87]. Jimenez et al. isolated the labeled *Enterococcus faecium* from the meconium of offspring after the oral inoculation of the strain with pregnant mothers. This indicated that the intestinal microorganisms of mothers potentially penetrated the placenta into the intestines of their offspring [58].

According to numerous authors, e.g., Gosalbes et al., the abundance of *E. coli* was confirmed in the meconium [65]. It was also the main factor contributing to the occurrence of early-onset sepsis in neonates with extremely low birth weight [84]. Aagaard et al. reported that the detection of commensal *Escherichia* in the meconium was associated with intrauterine colonization originating from the placenta [41]. According to Stoll et al., the acquisition of group B Streptococcus (GBS) and *E. coli* during delivery was identified as the main cause of EONS within 3 days after delivery in preterm infants [88]. In contrast, Dong et al. determined that skin or intestinal commensals, including *Staphylococcus* spp., *E. coli*, *K. pneumoniae*, or *Candida* spp., usually caused LONS [89]. Due to problems with the identification of a single causative agent, NEC was mainly associated with *Firmicutes* (coagulase-negative staphylococci) and *Proteobacteria* (*Cronobacter sakazakii*, *Klebsiella* sp., and *E. coli*) (Table 1) [88,89].

However, regarding significant correlations between the microbiota of the meconium and the duration of pregnancy, Ardissone et al. determined the relationships that were negatively linked to gestational age, with the exception of *Oxalicibacterium* [87]. The study revealed that the taxonomic families within the phylum Firmicutes that were correlated with gestational age included: *Bacillaceae*, *Staphylococcaceae*, *Enterococcaceae*, *Lactobacilaceae*, *Leuconostocaceae*, *Clostridiaceae*, *Peptostreptococcaceae*, *Veillonellaceae*, and *Erysipelotrichaceae*. At the genus level, the strongest correlations were assigned to *Enterococcus* and *Lactobacillus*, including an average of 8.67% versus 0.41% for *Enterococcus* and 0.82% versus 0.07% for *Lactobacillus* at <33 and >33 weeks, respectively. As regards *Actinobacteria*, *Bifidobacterium* was significantly correlated, with 5.47% versus 0.35% of all readings for <33 and >33 weeks, respectively. The phylum *Proteobacteria* that was significantly correlated with gestational age primarily included *Enterobacteriaceae*. The strongest correlations were identified for *Enterobacter* and *Photorhabdus*, with an average relative abundance of 6.35% versus 0.06% for *Enterobacter* and 0.98% versus 0.01% for *Photorhabdus* at <33 and >33 weeks, respectively. The abundance of *Tannerella* was lower and was correlated with low gestational age. Kang et al. determined that two classes, i.e., *Bacteroidetes* and *Firmicutes*, constituted the majority of the neonatal meconium microbiota. A significant increase in the relative abundance of *Bacteroidetes* and a decrease in *Proteobacteria* correlated with an increase in gestational age [90]. At the genus level, the dominant species included *Prevotella* and *Bacteroides*, with the relative abundance of *Prevotella* accounting for 20–30% of the intestinal microbiome. In healthy adults, two major classes, i.e., *Firmicutes* and *Bacteroidetes*, constituted over 90% of the gut microbiota, followed by *Actinobacteria* and Proteobacteria [91,92]. A study conducted in Germany by Klopp et al. also showed that gestational age was significantly linked to the meconium composition of extremely premature infants, and the most numerous phyla included *Firmicutes*, *Bacteroidetes*, *Proteobacteria*, and *Actinobacteria* (Table 2) [93]. 

The above research results, indicating a much lower diversity of bacterial populations, with an increased abundance within individual phyla, classes, and genera in preterm newborns, suggest a greater risk of the development of pathogenic flora through the dynamic multiplication and competitive displacement of protective microorganisms. Therefore, in combination with structural immaturity, the morbidity of newborns is inversely proportional to the age of pregnancy, i.e., the moment when significant environmental diversity becomes a protective factor, markedly contributing to the maintenance of the eubiotic state.

### 3.6. The Intestinal Microbiome in Newborns

**Figure 3:**
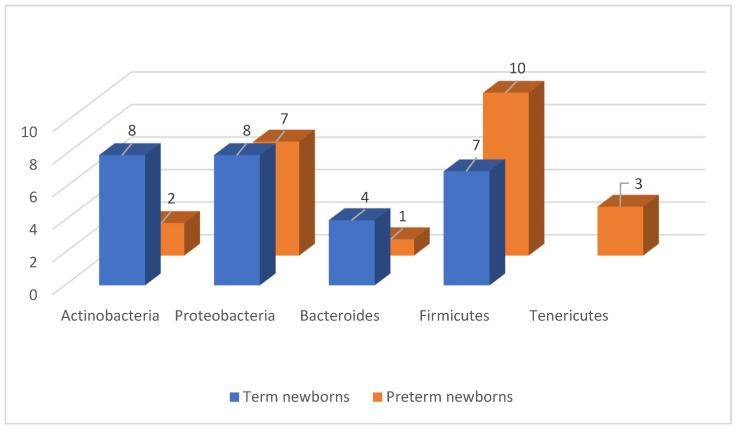
The diagram shows the types of bacteria that make up the gut microbiome according to their frequency of occurrence, divided into full-term and preterm newborns, as presented by the above-mentioned authors.

A difference between the intestinal microbiome of a term newborn and one born prematurely was noted when using material from newborns (Table 3 and Table 4) [9,14,41,42,68,86,87,94]. Sood et al. determined that in the first week of life, the intestinal microbiome of term newborns was largely colonized by *Actinobacteria* (including *Bifidobacterium*), *Proteobacteria*, *Bacteroides*, and, to a much lesser extent, by *Firmicutes* (including *Lactobacillus* spp., which are dominant in the vaginal flora) [42,70]. According to Aagaard et al., the phyla *Firmicutes* and *Tenericutes* and a much smaller abundance of *Actinobacteria* dominated in newborns with a body weight < 1200 g, which was consistent with the previously cited data on the microbiome of the placenta [41]. Arboleya et al. stated that preterm infants were characterized by an increased abundance of *Enterococcus*, *Enterobacter*, *Lactobacillus*, and *Staphylococcus* bacteria and a reduced abundance of *Bacteroides*, *Bifidobacterium,* and *Atopobium* compared to term infants [68]. They also showed that the colonization of the intestine by *Bifidobacterium* was delayed in preterm infants [95]. A prospective study showed that gestational age was significantly linked to intestinal colonization by *Bifidobacteria*. Delivery at the gestational age of <33 weeks appeared to impair bifidobacterial colonization and predispose premature infants to infections and intestinal diseases. Conversely, Bartnicka et al. reported that bacteria belonging to the genera *Bifidobacterium*, *Lactobacillus*, and *Streptococcus* dominated in term newborns, while *Enterobacteriaceae* and *Clostridium* dominated in premature infants (Figure 3) [9]. 

The above data are summarized in Table 5.

Based on the analysis, a correlation between the intestinal microbiome of a newborn may be concluded, taking account of the birth age, and the potential microbiome of the mother, broken down by the site of colonization (Table 6 and Table 7).

It is obvious that the mode of delivery, activities and drugs used perinatally, and the first contact with the external environment postnatally are important factors regulating the composition of the intestinal microbiota. 

### 3.7. Influence of the Intrauterine Microbiome on the Child’s Immune System

The immune aspect may not be ignored. It was confirmed that the microbiota of the mother’s vagina during pregnancy influenced the immunity of the fetus in the uterus, even before passing through the vaginal canal during childbirth. Over the past decade, studies involving both the 16S and metagenomics have shown that the human vagina mainly contains *Lactobacillus* spp. at concentrations reaching 10^7^–10^9^ per gram of vaginal secretion [77,96,97]. The resultant state of eubiotics is a protective factor against other species due to the competitive exclusion concept. 

A pilot study conducted in 2016 showed that the percentage of pregnancies decreased by almost 40% in women without the dominance of *Lactobacillus* (non-*lactobacillus*-dominant, NLD) species in the uterus [74]. Recent research has suggested that an inflammatory response may be triggered in the endometrium by NLD-phenotype microbiota, affecting the success of embryo implantation. It may result from the regulation of inflammatory mediators during blastocyst adhesion to the epithelial wall of the endometrium [29]. Research showed that *Lactobacillus* bacteria were more prominent in women with endometrial polyps or chronic endometritis [78]. Conversely, chronic endometritis was found to be associated with recurrent pregnancy loss and the presence of plasma cells in endometrial biopsy [98,99,100,101]. Abundant bacteria were detected in such patients, including *Neisseria gonorrhea*, *Chlamydia trachomatis*, *Escherichia coli*, *Streptococcus*, *Staphylococcus*, and *Enterococcus faecalis*. Non-microbiological causes were also identified. It was also hypothesized that endometriosis changed the endometrium through the intensification of inflammation and resistance to progesterone. This might affect implantation and increase the risk of miscarriage and pregnancy complications, e.g., pregnancy-induced hypertension and preterm birth [102]. 

Neonates whose mothers were intragestationally vaginally colonized with *Lactobacillus* had a higher percentage of CD45RO+ cells and reduced IL-12 in the umbilical cord blood. Therefore, the authors stated that lactobacilli in the mother’s vagina influenced the development of fetal immunity [10,103].

The microflora during pregnancy initiates the immune programming of the offspring in various, interrelated ways that are not necessarily mutually exclusive. The above was confirmed by research by Hu et al., Madan et al., and Ardissone et al., who showed that the intestines of the fetus were colonized by bacteria during pregnancy [14,86,87]. Aagaard et al. and Collado et al. demonstrated that the placenta contained a unique microbiome [41,94]. Conversely, Ferretti et al. noted a greater stability of maternal intestinal strains in the intestines of newborns compared to vaginal and dermal strains [104]. The majority of authors indicated a disorder related to *Proteobacteria* and *Firmicutes* prior to the occurrence of NEC. Research also revealed that the diversity of gut bacteria was reduced in preterm infants at risk of LONS and NEC [11]. However, chorioamnionitis and fetal inflammatory response syndrome were linked to the development of numerous diseases in premature infants, i.e., cerebral palsy [105,106], intraventricular hemorrhage (IVH) [107,108,109], retinopathy of prematurity (ROP) [110], NEC [108,111] and EONS [109,112,113]. At the same time, it was shown that fetal membrane inflammation played a protective role in LONS by accelerating the maturation of the immune system [113].

The development of atopic disease in the offspring of mothers who used antibiotic therapy during pregnancy and, thus, modeled the maternal microflora (along with its metabolites) may be the evidence of the impact of this process on the modeling of the immune response [114].

During the first postnatal week, an increased abundance of the genera *Bacteroides* and *Bifidobacterium* was found in term newborns delivered vaginally [115,116]. *Bifidobacterium* and *Bacteroides* are genera that are thought to promote health and interact directly with the developing immune system during infancy [117,118,119]. It was found that *Bacteroides fragilis* produced a bacterial polysaccharide that assisted in the maturation of the immune system and the production of regulatory T lymphocytes [118,119,120]. A study conducted on the murine model, which is known for intestinal barrier defects, revealed that oral treatment with *B. fragilis* reduced intestinal permeability and changed the microbiological composition [121]. Research on the effect of antibiotic therapy on bacterial flora showed that early antibiotic use was associated with the reduced abundance of *Bifidobacterium* and *Bacteroides* in preterm infants. Therefore, it was suggested that early exposure to antibiotics might affect the future composition of intestinal bacteria in preterm infants. 

Arboleya et al. demonstrated that antibiotics used perinatally affected the quantitative and qualitative structure of the intestinal microbiota. It was particularly manifested as the increased abundance of bacteria from the family *Enterobacteriaceae* and *Clostridiaceae* and the reduction in protective *Bifidobacterium* and *Lactobacillus* in infants [122].

This is of key importance from the viewpoint of the susceptibility to infections and their severity. At the same time, it translates into psychophysical development and metabolic disorders, which may predispose to the development of civilization diseases exacerbated by environmental factors.

### 3.8. Pregnancy Complications—Pre-Eclampsia and Its Spectrum

The participation of systemic inflammatory response in pregnancies complicated by pre-eclampsia (PEC4) and intrauterine growth restriction (IUGR) led to the development of a hypothesis that maternal infections might be an important factor in the pathogenesis of pregnancy complications. 

It was also demonstrated that the risk of pre-eclampsia was increased in women with asymptomatic bacteriuria, urinary tract infection, and chronic pyelonephritis [123,124]. Den Hollander et al. confirmed that *Helicobacter pylori*, being a cause of chronic inflammation, was associated with an increased risk of PEC [125]. Moreover, Li Juan et al. demonstrated that pre-eclampsia was associated with a disturbance in the composition of intestinal microflora compared to women whose pregnancies were uncomplicated [126]. Dunn et al. published a review of a possible relationship between the microbiome and the development of pre-eclampsia, which showed that the PEC microbiome was studied by five groups of researchers [127]. Two studies analyzed placental location, and the remaining three examined the oral cavity, intestine, or intra-amniotic site. Some findings confirmed the link between pathogenic bacteria and PEC. However, specific pathogenic organisms were identified, so further research is justified. In a study conducted in 2015, placental tissue samples were collected from women with and without pre-eclampsia and tested for the presence of bacteria with the use of next-generation sequencing and PCR for 16s rRNA. A total of 12.7% of the tissues of women with PEC tested positive in the PCR test, while all the placentas in the control group tested negative [128].

In contrast, a study in which researchers carefully controlled for possible contaminants using multiple detection methods, including culture, qPCR, 16S rRNA gene sequencing, and shotgun metagenomics, revealed that no placental microbiota could be identified in the placenta.

## 4. Conclusions

The present review on the microbiome of both term newborns and premature infants indicates a possible intrauterine colonization of the fetus depending on the duration of pregnancy. The colonization occurs both via the vaginal and intestinal route (hematogenous route). The postnatal examination of the neonatal meconium clearly confirms the above hypothesis, especially if we consider the duration of pregnancies (the meconium of premature versus term neonates) and the continuity of fetal membranes. However, we noted differences in the demonstrated representatives of various types of bacteria, phyla Firmicutes and Actinobacteria in particular, taking account of the distribution in their abundance in individual groups of pregnancy duration. Simultaneously, the distribution of the phyla Actinobacteria and Proteobacteria was consistent. However, as regards the occurrence of specific species and genera, their similarity should be emphasized. Considering the duration of pregnancy, it can also be concluded that the bacterial flora of vaginal origin dominates in preterm newborns, while the flora of intestinal origin dominates in term newborns. This might explain the role of bacterial and infectious factors in inducing premature birth with the rupture of fetal membranes. The above conclusions suggest that appropriate shaping of the mother’s bacterial flora, comprising the microbiome of the digestive and the genital tract during pregnancy, may contribute to the prevention of premature births with the proper shaping of the fetal intestinal microbiota. This is an extremely important aspect, thanks to which it would be possible to avoid obstetric failures and, at the same time, influence the life and development of a person from conception to old age. This also translates into the financial aspect of the functioning of health care and the costs associated with the treatment of immunological and metabolic diseases associated with disturbed intestinal microbiota from the moment of conception. Our publication aimed to show this extremely important aspect, which obviously requires further research. Such research would constitute the basis for creating a scheme of action for the proper shaping of the microbiota of a pregnant woman.

## Figures and Tables

**Table 1:** Microbiota differences depending on diseases.

	Disease	NEC	EONS	LONS
Organism				
*Escherichia coli*		+	+	+
*Streptococcus agalactiae*			+	
*Staphylococcus* spp.		+		+
*Klebsiella pneumoniae*		+		+
*Cronobacter sakazakii*		+		
*Candida* spp.				+

**Table 2:** Correlation between meconium microbiota and the duration of pregnancy.

Researcher		Ardissone et al. [87]	Kang et al. [90]	Klopp et al. [93]
Time of Delivery	Phylum	Occurrence		
<33 weeks	*Firmicutes*	8.67% *Enterococcus* 0.82% *Lactobacillus*	most of the neonatal meconium microbiota; the dominant ones: *Prevotella* *Bacteroides*	detected
	*Actinobacteria*	*Bifidobacterium* 5.47%	-	detected
	*Proteobacteria*	6.35% *Enterobacter* 0.98% *Photorhabdus*	detected	detected
	*Tannerella*	low bacterial count	*-*	-
	*Bacteroidetes*	-	most of the neonatal meconium microbiota	detected
>33 weeks	*Firmicutes*	0.41% *Enterococcus* 0.07% *Lactobacillus*	most of the neonatal meconium microbiota	*-*
	*Actinobacteria*	0.35% *Bifidobacterium*	*-*	*-*
	*Proteobacteria*	0.06% *Enterobacter* 0.01% *Photorhabdus*	lower compared to newborns delivered at lower gestational ages	*-*
	*Tannerella*	very low bacterial count	*-*	*-*
	*Bacteroidetes*	*-*	higher compared to newborns delivered at lower gestational ages; the dominant ones: *Prevotella* *Bacteroides*	*-*

“-”—non-occurrence.

**Table 3:** The intestinal microbiome in term newborns.

	Researcher	Sood et al. [42]	Bartnicka et al. [9]	Ardissone et al. [87]	Collado et al. [94]	Jianzhong Hu et al. [86]
Taxa						
*Actinobacteria*		++ (including *Bifidobacterium*)	++		++ *Propionibacterium*	++
*Proteobacteria*		++		++ *Stenotrophomonas*, *Escherichia*	++ *Enterobacteriaceae*, *Escherichia*, *Shigella*	++
*Bacteroides*		++				++
*Firmicutes*		+ (including *Lactobacillus*)	++ *Lactobacillus*, *Streptococcus*		++ *Streptococcus*, *Lactobacillus*	++

The number of “+” signs is proportional to the size of the microbial population.

**Table 4:** The dominant intestinal microbiome of preterm infants.

	Researcher	Arboleya et al. [68]	Bartnicka et al. [9]	Aagaard et al. Intestinal Microbiome of a Newborn with the Body Weight < 1200 g [41]	Ardissone et al. [87]	Juliette Madan et al. [14]
Taxa						
*Actinobacteria*				+	+	
*Tenericutes*				++	+	
*Firmicutes*		++ *Enterococcus* *Lactobacillus* *Staphylococcus*	++ *Clostridium*	++	++	++ *Lactobacillus* *Staphylococcus*
*Proteobacteria*		++ *Enterobacter*	+ *Enterobacteriaceae*		++	++ *Enterobacteriales*
*Bacteroidetes*					+	

The number of “+” signs is proportional to the size of the microbial population.

**Table 5:** The diversity of the microbiota in both full-term and premature infants.

	Full-Term versus Premature Newborn	The Intestinal Microbiome in Term Newborns	The Intestinal Microbiome in Term and Preterm Newborns	The Intestinal Microbiome in Preterm Newborns
Phylum				
*Firmicutes*		*Streptococcus*	*Lactobacillus*	*Staphylococcus* *Enterococcus* *Clostridium*
*Proteobacteria*		*Escherichia* *Shigella* *Stenotrophomonas*	*Enterobacteriaceae*	*Enterobacter*
*Actinobacteria*		*Bifidobacterium* *Propionibacterium*	The authors did not perform species differentiation	insignificant amount
*Bacteroidetes*		insignificant amount	The authors did not perform species differentiation	trace amount
*Tenericutes*		lack	The authors did not perform species differentiation	insignificant amount

**Table 6:** Neonatal gut microbiome vs. potential microbiome of the gut, vagina, amniotic fluid, and placenta of the mother—term newborns.

	Site	Intestine	Vagina	Amniotic Fluid and Placenta
Organism				
*Firmicutes*		++ including *Streptococcus*	+ including *Lactobacillus*	+ *Lactobacillus*, *Streptococcus*
*Tenericutes*		++		
*Proteobacteria*		++ including *E. Coli* (+++), non-pathogenic *Neisseria* species		++ *Escherichia/Shigella*, *Enterobacter*
*Bacteroidetes*		++ including *Prevotella tannerae*	++	
*Fusobacteria*		++		
*Actinobacteria*		++ including *Bifidobacterium*		++ *Propionibacterium*

The number of “+” signs is proportional to the size of the microbial population.

**Table 7:** Neonatal gut microbiome vs. potential microbiome of the gut, vagina, amniotic fluid, and placenta of the mother—preterm newborns.

	Site	Intestine	Vagina	Amniotic Fluid and Placenta
Organism				
*Firmicutes*		+++ *Enterococcus*, *Staphylococcus*, *Clostridium*	+++ including *Lactobacillus*, *Staphylococcus*	++
*Proteobacteria*		+++ *Enterobacter*		++
*Bacteroidetes*		+	+	+
*Actinobacteria*		+ *Bifidobacterium*	+ *Atopobium*	+

The number of “+” signs is proportional to the size of the microbial population.
